# Interactions of free-living amoebae with the rice fungal pathogen, *Rhizoctonia solani*

**DOI:** 10.1186/s13104-019-4802-2

**Published:** 2019-11-15

**Authors:** John J. Long, Emily K. Luna, Mary Jackson, William Wheat, Courtney E. Jahn, Jan E. Leach

**Affiliations:** 1 Department of Plant Pathology, The Ohio State University, Columbus, OH USA; 2 Department of Bioagricultural Sciences and Pest Management, Colorado State University, Fort Collins, CO USA; 3 Mycobacteria Research Laboratories, Department of Microbiology, Immunology, and Pathology, Colorado State University, Fort Collins, CO USA

**Keywords:** Free-living amoebae, *Rhizoctonia solani*, Sheath blight, *Acanthamoeba*, *Dictyostelium*, *Vermamoeba*

## Abstract

**Objective:**

*Rhizoctonia solani* is a soil-borne fungal pathogen of many important crop plants. In rice, *R. solani* causes sheath blight disease, which results in devastating grain yield and quality losses. Few methods are available to control this pathogen and classic single gene resistance mechanisms in rice plants have not been identified. We hypothesize that alternate means of control are available in the environment including free-living amoebae. Amoebae are soil-, water- and air-borne microorganisms that are predominantly heterotrophic. Many amoeba species are mycophagous, and several harm their prey using mechanisms other than phagocytosis. Here, we used light and scanning electron microscopy to survey the interactions of *R. solani* with four amoeba species, with the goal of identifying amoebae species with potential for biocontrol.

**Results:**

We observed a wide range of responses during interactions of *R. solani* with four different free-living amoebae. Two *Acanthamoeba* species encyst in co-cultures with *R. solani* at higher rates than medium without *R. solani*. *Vermamoeba vermiformis* (formerly *Hartmanella vermiformis*) attach to *R. solani* mycelium and are associated with mycelial shriveling and perforations of fungal cell walls, indicating an antagonistic interaction. No phenotypic changes were observed in co-cultures of *Dictyostelium discoideum* and *R. solani*.

## Introduction

*Rhizoctonia solani* is a significant fungal pathogen of rice that is particularly difficult to control. This fungus causes rice sheath blight, a necrotic disease characterized by lesions initiating at the apical ends of rice sheaths. As a persistent and virulent soil-borne pathogen, *R. solani* can reduce yields by half in conducive environmental conditions [1]. The fungus survives in soil and in fields by forming sclerotia, which are condensed bodies of fungal hypha that may survive in soil for up to 2 years [1, 2]. The broad host spectrum of *R. solani* allows the fungus to infect alternative hosts as another means to remain in an environment [3]. Compounding the issue of pathogen persistence is that there are currently no plant disease resistance genes identified for control of *R. solani*, although potential quantitative trait loci that incrementally increase plant resistance have been identified [1, 4, 5]. To reduce instances and severity of outbreaks from *R. solani*, additional methods of control are needed. To that end, we explored interactions of the fungus with free-living amoebae, with a view towards adapting amoebae antagonistic to *R. solani* as biological control agents.

Historical studies of the interactions of free-living amoebae and *R. solani* are limited. In one study, sclerotia and hyphae inoculated into soil samples showed signs of extensive predation by mycophagous protozoa [6]. Amoebae recovered from the soil were identified to be a species of *Thecamoeba* based on morphology.

Interactions among other fungi and amoebae have been well described. After incubation in soil, conidia of *Cochliobolus sativus* were lysed, with multiple perforations visible in the cell wall, suggestive of amoebal predation [7]. Some amoebae engulf entire conidia, after which they encyst, slowly digesting their prey inside the cyst [7, 8]. A member of the genus *Acanthamoeba* also preys on a variety of fungi pathogenic to mammals, such as *Blastomyces dermatitidis* and *Cryptococcus neoformans* [9]. Interestingly, some strains of *C. neoformans* survive inside *A. castellanii,* and use the amoebae as a reservoir for future infections [9].

Given their proximity in the phytobiome, we hypothesize that free-living amoebae interact with *R. solani*. To study these interactions, we observed different amoebal species (*A. castellanii*, *A. polyphaga*, *D. discoideum* and *V. vermiformis)* after co-culture with *R. solani* by light and fluorescence microscopy. Of the four amoebae tested, we found that only *V. vermiformis* caused detrimental changes in the fungal hyphae, and we further explored these interactions with scanning electron microscopy (SEM).

## Main text

### Methods

#### Amoebae and fungi culturing conditions

*Acanthamoeba* species were cultured at 28 °C in a modified peptone, yeast and glucose medium (PYG), *V. vermiformis* was cultured at 28 °C in a modified peptone, yeast extract, liver digest, hemin and serum medium (PYNFH) and *D. discoideum* was maintained at room temperature in a modified rich axenic medium (HL5) [10]. Amoebae cultures were inoculated from frozen stocks into 100 × 15 mm petri dishes with 30 mm walls holding 10 mL of medium supplemented with Gibco penicillin/streptomycin (Invitrogen; California, United States) to a 1× working concentration. Once initial cultures reached turbidity, *Acanthamoeba* species and *V. vermiformis* were passaged every 5 days by transferring 500 µL of culture into 10 mL of fresh medium. *D. discoideum* was passaged every 3 days. Amoebae cultures were passaged no more than three times prior to use in our studies.

*Rhizoctonia solani* was cultured on 1/2 strength potato dextrose agar (PDA; Difco) from frozen stocks prepared on barley seeds according to [11]. Initial cultures were incubated at 22 °C with 16 h of light for 10 days, then stored at 4 °C for use as a source of agar plugs. Source plates were kept for up to 3 weeks before starting new cultures from stock. Agar plugs 7.5 mm in diameter were subcultured onto autoclaved cellophane overlaid onto 1/2 strength PDA and incubated for 7–10 days at the above conditions before use in experiments.

#### Co-cultures of amoebae and *R. solani*

Confluent cultures of amoeba were starved overnight in diluted medium at the temperatures described above, except for *D. discoideum*, which was kept in full strength medium. *Acanthamoeba* were starved in 1/5 strength PYG while *V. vermiformis* were starved in 1/2 strength PYNFH; media were diluted using Page’s modified amoeba saline (PAS) [10]. Amoebal cell density was calculated using a direct cell counting method involving trypan blue exclusion and a hemocytometer. Only cultures with over 90% viable trophozoites were used. Amoebae cultures were adjusted to concentrations of 2 × 10^5^ cells/mL in fresh, diluted medium.

Plugs of fungal mycelia were cut with a sterilized borer with an internal diameter of 5 mm. Fungal plugs were removed from the agar plate using sterile forceps and rinsed once in sterile, distilled water and transferred to a 1.5 mL centrifuge tube. 500 μL of amoebae culture was added to each tube containing fungi. Each amoebae and fungal combination were prepared in triplicate for sampling at each time point of 0, 24, and 48 h. Co-cultures with *Acanthamoeba* sp. or *V. vermiformis* were incubated at 28 °C and cultures with *D. discoideum* were incubated at 22 °C.

#### Microscopy

Co-cultures were centrifuged at 150 × G for 3 min and the supernatant was removed. Pellets were washed three times with 500 μL of PAS by centrifugation at 150 × G for 3 min each time. After washing, pellets were fixed with 100 μL of 4% paraformaldehyde for 48 h. After fixation, samples were pelleted and then suspended in 30 μL of PAS. For viability staining, samples were first dyed with 4 μL of 8 mg/mL fluorescein diacetate (FDA) and 25 μL of 2 mg/mL propidium iodide (PI) for 15–20 min in the dark. Samples were then washed and fixed as noted above, then mounted on glass microscope slides.

Standard light and fluorescence microscopy were conducted on a Zeiss Axioskop microscope fitted with Chroma Technology filters. FDA was visualized using 480 and 535 nm excitation and emission filters, respectively, and PI was visualized with 535 nm excitation and 610 nm emission filters. Images were captured and false-colored using the Prog Res Capture Pro software (Jenoptik) and multi-color images were obtained by overlaying images from the FDA and PI channels. Adobe Photoshop CS6 was used to crop and adjust images.

Confocal laser scanning microscopy was carried out on a Zeiss LSM 510 inverted microscope. Samples were excited with a 488 nm laser and emission filters were set to 480 and 590 nm for FDA and PI, respectively. At 400× and 630× magnification, three random fields were taken per sample and images were taken at ten different depths in 0.5–1.5 µm increments. Images were merged into one using the Zeiss Zen 2009 software.

#### Scanning electron microscopy

Co-cultures were prepared such that (1) the two organisms could directly contact one another or (2) the two species were prevented from physical contact. In the former, amoebae were adjusted to a concentration of 2 × 10^5^ trophozoites/mL and 10 mL of the culture was added to a high-walled petri dish. Fifteen plugs of *R. solani* were added to the culture and the dish was sealed with Parafilm then stored in a plastic bag. To separate the two species, co-cultures of *V. vermiformis* and *R. solani* for SEM were prepared following a modified procedure from Homma and Ishii [6]. Two nucleopore membranes (25 mm in diameter; Whatman #110610; Maidstone, United Kingdom) with 1.0 µm pores were used to sandwich three fungal discs, the edges of the membrane sandwiches were sealed with silicon vacuum grease. Five sandwiched membranes were added to a petri dish containing 2 × 10^6^ *V. vermiformis* trophozoites at 10 mL of final volume. Co-cultures were incubated at 22 °C with 16 h of light. At 0, 2, 6, 12, and 24 h, three disks from each culture were transferred to individual micro-centrifuge tubes and centrifuged once at 150 × G for 3 min. Supernatant was discarded and samples were washed once in 500 μL PAS. After centrifugation and removal of the wash, samples were fixed in 2.5% glutaraldehyde buffered in 0.15 M Sorensen’s phosphate buffer, pH 7.0 (22 °C for 30–60 min, followed by 4 °C). Tissue samples were dehydrated through a graded ethanol series, followed by final dehydration using a BioRad E3000 critical point dryer (Quorum Technologies, East Sussex, England). All samples prepared for SEM were sputter coated with 10 nm gold, imaged at 5 kV with a JEOL JSM-6500F Field Emission Scanning Electron Microscope. All images were captured as tiff files.

### Results with discussion

#### Microscopy reveals a diverse array of reactions

**Fig. 1:**
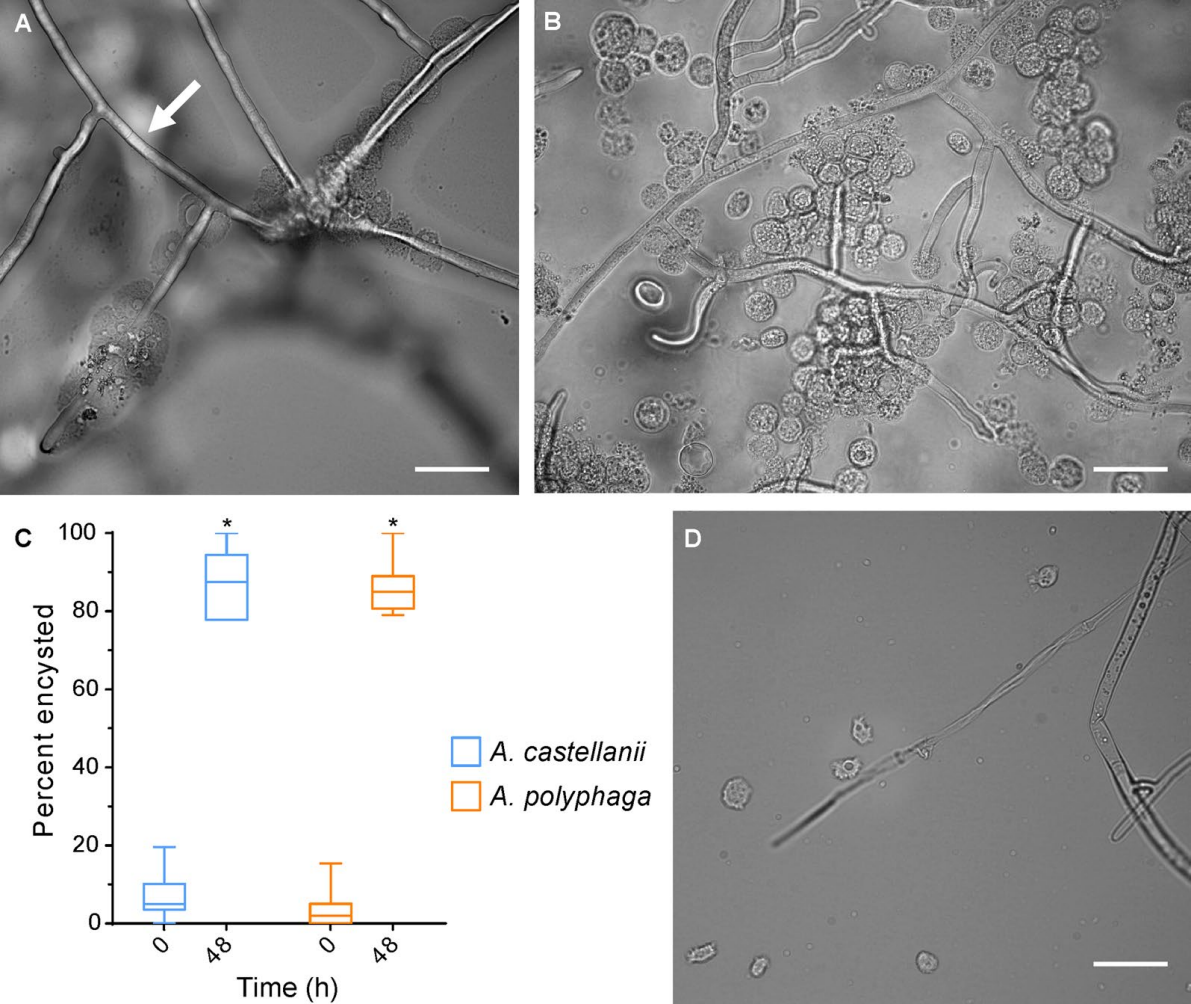
Light microscopy of amoeba and *R. solani* co-cultures. **a** *A. castellanii* and *R. solani* after 48 h. **b** *A. polyphaga* and *R. solani* after 24 h. The smooth and straight cell walls of *R. solani* are easily visible, indicated by a white arrow. **c** Encystment of *A. castellanii* and *A. polyphaga* after co-culture with *R. solani*, stars denote p < 0.0001 calculated by a Student’s *T* test. **d** *D. discoideum* and *R. solani* after 24 h. All images were taken at ×630 magnification and scale bars are 10 µm

Different genera of amoebae trophozoites interacted differently with *R. solani* mycelium in co-cultures. At 48 h, the two *Acanthamoeba* species encysted at higher rates than the amoebae-only control in PAS non-nutrient media (Fig. 1a–c). The cysts clumped together around the mycelium rather than floating free in the culture, an observation noted in co-cultures prepared with and without centrifugation. No changes in fungal mycelium were observed after co-cultivation. The hyphae remained intact with no visible perforations. Nuclei, stained red with propidium iodide, were not disrupted. In addition, the hyphal cell wall remained smooth and mycelia were branched at right angles, as is typical for *R. solani*.

*Dictyostelium discoideum* did not have any apparent physical interaction with the fungal hyphae. Under light microscopy, the amoebae did not attach to hyphae or form sporulating bodies, a sign of nutrient deprivation or environmental incompatibility. The fungal hyphae were not visibly altered, and mycelium remained intact and with no observed perforations (Fig. 1d).

**Fig. 2:**
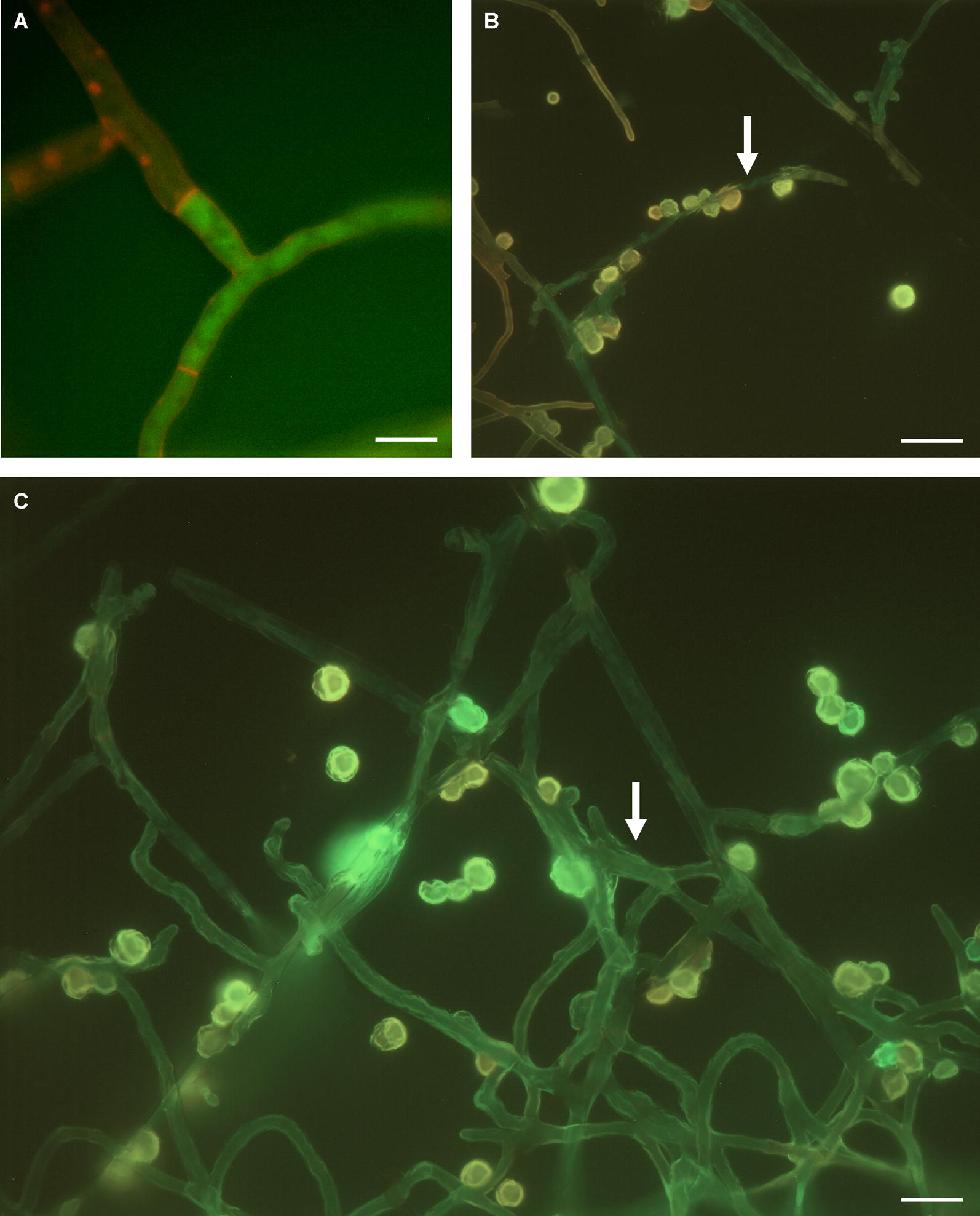
Fluorescence microscopy of *V. vermiformis* and *R. solani* after co-cultivation. **a** *R. solani* without amoeba, imaged at 24 h, showing the smooth hyphal surfaces and right-angle branching of mycelia. **b** Co-culture at 24 h showing the shriveled morphology and physical association of amoebae and fungal hyphae, denoted by white arrows. **c** Fluorescence image, with the emission of FITC (green) and PI (red) overlaid, shows both amoeba trophozoites and fungal hyphae are alive. Scale bars are 10 µm

**Fig. 3:**
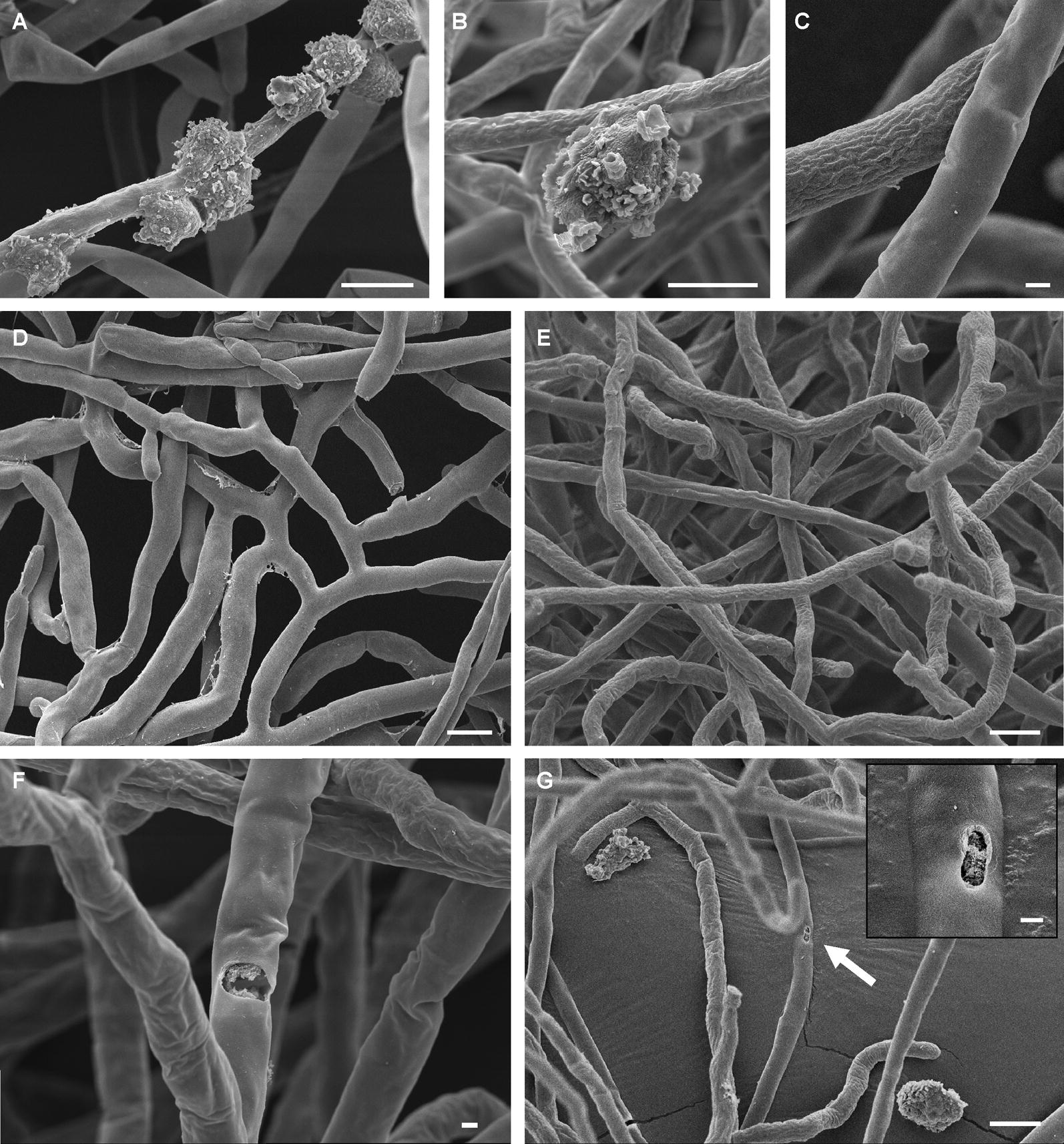
Scanning electron microscopy of *V. vermiformis* and *R. solani* after co-culture. Organisms were not separated by a nucleopore membrane; 24 h co-culture. **a** Micrographs show *V. vermiformis* trophozoites partially wrapped around *R. solani* hyphae. **b** A close-up image of a trophozoite and shriveled hypha. **c** A close-up micrograph of hyphae at varying intensities of shriveling. Organisms were separated by a nucleopore (1 µm) membrane and evaluated at 0 (**d**) and 24 h (**e**) after co-cultivation. The hyphae of *R. solani* develop a shriveled exterior even when separated from the amoeba after 24 h. **f**, **g** A perforation with smooth edges detected on the hyphae after co-culturing with amoebae in the same medium. Scale bars in **a**, **b**, **d**, **e** and **g** are 10 µm; scale bars in **c**, **f** and inset of **g** are 1 µm

Of the four amoebae tested, only *V. vermiformis* had a noticeable effect on the fungus. Trophozoites were physically attached to the mycelium and remained viable for at least the 24 h of co-culture (Fig. 2). Scanning electron microscopy revealed that, after 24 h co-cultivation, the surface of *R. solani* mycelia became mottled and shriveled (Fig. 3a–c). The shriveling was not observed when the fungus was cultured in PYNFH medium without *V. vermiformis* (Fig. 3d). The hyphal appearance after co-cultivation with *V. vermiformis* was in stark contrast from the smooth cell walls and branching hyphae of normal *R. solani* [12]. Interestingly, the shriveled appearance of hyphae occurred even when *V. vermiformis* and *R. solani* were physically separated by membranes (Fig. 3e–g). While transient contact can occur through the 1 µm pores of the membrane, the amoebae cannot physically wrap around the mycelium. Perforations with smooth and rounded edges, although rare, were detected on the fungal mycelium of the physically separated co-cultures.

It is not surprising that the panel of amoebae presented a variety of interactions with the fungi. Some bacterial species are known to antagonize amoebae and force encystment or even lyse the amoebae [13–15]. The cause of encystment in *Acanthamoeba* is not known at this time, and we have not ruled out nutrient deprivation as the factor. To our knowledge, there are no characterized mechanisms of anti-amoebal activity from *R. solani*. The reason for and the nature of the *Acanthamoeba* cysts’ physical association with the fungal hyphae is not understood. One explanation could be that *Acanthamoeba* trophozoites attach and attempt to feed, and in doing so encounter something that stimulates encystment.

The shriveling response of the fungi after exposure to *V. vermiformis* suggests antagonism. Because the same response occurred with and without direct contact of the two organisms, it is possible the fungi is reacting to a secreted amoebal factor. The extent of the response is still uncharacterized and the effects of amoebal secretions on fungi are not well studied. However, some plant extracts are capable of causing similar responses in *R. solani* [12, 16]. In those cases, shriveling of *R. solani* after contact with plant extracts was caused by collapse of the cell wall. Lastly, the observations of perforations of hyphae, although rare, in the membrane-separated cultures suggest that *V. vermiformis* can puncture *R. solani* cell walls. In cultures not separated by a membrane, it is possible that perforations are obscured by an attached amoeba.

## Limitations

1. The encystment of *Acanthamoeba* species early in interactions with *R. solani* may or may not have required fungal contact.
2. The presence of *V. vermiformis* trophozoites attached to mycelia in the SEM studies may have obscured observation of perforations in fungal cell walls.

## Data Availability

All data generated or analyzed during this study are included in this published article.

## Abbreviations

SEM: scanning electron microscopy
PYG: modified peptone, yeast and glucose medium
PYNFH: modified peptone, yeast extract, liver digest, hemin and serum medium
HL5: modified rich axenic medium
PDA: potato dextrose medium
PAS: Page’s modified amoeba saline
FDA: fluorescein diacetate
PI: propidium iodide
